# An autocrine sphingosine-1-phosphate signaling loop enhances NF-κB-activation and survival

**DOI:** 10.1186/1471-2121-11-45

**Published:** 2010-06-24

**Authors:** Tomas Blom, Nina Bergelin, Annika Meinander, Christoffer Löf, J Peter Slotte, John E Eriksson, Kid Törnquist

**Affiliations:** 1 Department of Biology, Åbo Akademi University, 20520 Turku, Finland; 2 Department of Biochemistry and Pharmacy, Åbo Akademi University, 20520 Turku, Finland; 3 Turku Centre for Biotechnology, Åbo Akademi University & University of Turku, 20520 Turku, Finland; 4 The Minerva Foundation Institute for Medical Research, 00290 Helsinki, Finland; 5 Current Address: Institute of Biomedicine, University of Helsinki, 00290 Helsinki, Finland; 6 Current Address: Breakthrough Toby Robbins Breast Cancer Research Centre, Institute of Cancer Research, London SW3 6JB, UK

## Abstract

**Background:**

Sphingosine-1-phosphate (S1P) is a bioactive lipid that regulates a multitude of cellular functions, including cell proliferation, survival, migration and angiogenesis. S1P mediates its effects either by signaling through G protein-coupled receptors (GPCRs) or through an intracellular mode of action. In this study, we have investigated the mechanism behind S1P-induced survival signalling.

**Results:**

We found that S1P protected cells from FasL-induced cell death in an NF-κB dependent manner. NF-κB was activated by extracellular S1P via S1P_2 _receptors and G_i _protein signaling. Our study also demonstrates that extracellular S1P stimulates cells to rapidly produce and secrete additional S1P, which can further amplify the NF-κB activation.

**Conclusions:**

We propose a self-amplifying loop of autocrine S1P with capacity to enhance cell survival. The mechanism provides increased understanding of the multifaceted roles of S1P in regulating cell fate during normal development and carcinogenesis.

## Background

Sphingolipids regulate cellular processes such as migration, survival and differentiation [1,2]. Sphingosine-1-phosphate (S1P), the most extensively studied of the bioactive sphingolipids, acts as a high affinity agonist at five known G protein-coupled receptors named S1P_1_-S1P_5 _[3]. The S1P-receptors are important for regulating cell migration [4-6], proliferation and survival [7]. In addition, it has been shown that S1P can act intracellularly as a calcium releasing second messenger [8,9] and as a regulator of histone acetylation and transcription [10]. It is likely that some effects attributed to intracellular S1P can also be explained by signaling through internalized G protein-coupled receptors [11,12].

S1P is synthesized through sphingosine kinase (SphK) -catalyzed phosphorylation of sphingosine. Type 1 sphingosine kinase (SphK1) is generally associated with cell survival, and several mechanisms for regulating its function have been identified. Growth factors [13,14], cytokines [15,16], and even S1P itself [17,18] have been shown to stimulate SphK-activity and S1P-production. ERK1/2 mediated phosphorylation on Ser225 directly activates SphK1, and this is also a prerequisite for the translocation of SphK1 to the plasma membrane [19]. Furthermore, binding to Ca^2+^-calmodulin has been shown to be crucial for translocation of SphK1 to the plasma membrane [20,21]. SphK1 may also be regulated by lipids such as phosphatidylserine [22] or phosphatidic acid [23].

An increase in SphK1-activity often correlates with enhanced survival and proliferation. Several studies have shown that intracellular S1P is exported and acts on G protein coupled S1P-receptors to induce survival signaling [24-26]. SphK1 itself may also be exported from cells and retain its catalytic function in the extracellular space [27,28], thus synthesizing S1P that has access to S1P-receptors in the plasma membrane.

In this study, we have investigated the signaling mechanisms activated by exogenous S1P, and in particular the effects of the subsequent increase in cellular S1P-production. We found that S1P mediated protection from death receptor-induced apoptosis in an NF-κB dependent manner. Intriguingly, exogenously added S1P induced several cell types to synthesize and secrete additional S1P. The S1P that is secreted from cells can further enhance NF-κB activation through G protein coupled S1P-receptors. We demonstrate here that a G protein coupled receptor agonist can induce its own production and secretion at physiologically relevant levels.

## Methods

### Materials

Fluo-3 AM and BAPTA AM were purchased from Molecular Probes (Eugene, OR, U.S.A.). D-*erythro*-sphingosine-1-phosphate, D-*erythro*-N,N-dimethylsphingosine, dihydro-sphingosine-1-phosphate, and GF109203× were from Biomol (Plymouth meeting, PA, U.S.A.) and D-*erythro*-sphingosine from Sigma (St. Louis, MO, U.S.A.). Phorbol 12-myristate 13-acetate (PMA), the sphingosine kinase inhibitor 2-(*p*-Hydroxyanilino)-4-(*p*-chlorophenyl)thiazole (SKi), PD98059, Bay 11-7082, and Wortmannin were from Calbiochem (Darmstadt, Germany). [^3^H]-sphingosine was from NEN Life Science Products (Boston, MA, U.S.A.). U73122 and Pertussis toxin were purchased from Sigma (St Louis, MO, U.S.A.). VPC 23019 was from Avanti (Alabaster AL, U.S.A.). FLAG-tagged TRAIL and SuperFasLigand were from Alexis (San Diego, CA, U.S.A.). TRAIL was crosslinked with M2 anti-FLAG antibody (Sigma, St. Louis, MO, U.S.A.) prior to stimulating cells. The S1P_2,4,5 _antibodies were from Santa Cruz Biotechnology (Santa Cruz, CA, U.S.A.), and the S1P_1,3 _antibodies were from both Santa Cruz and Cayman Chemicals (Ann Arbor, MI, U.S.A.). Pre designad SMARTpool siRNA's were purchased from Dharmacon (Lafayette, CO, U.S.A.).

### Cell culture

HeLa cells and MEL-7 cells were cultured in Dulbecco's modified Eagle's medium supplemented with 10% fetal bovine serum, 2 mM L-glutamine, and 50 U/ml penicillin and 50 μg/ml streptomycin at 37°C in a water-saturated atmosphere of 5% CO_2 _and 95% air. Cells were cultured in medium with serum replaced by 0.2% fatty acid free BSA 24 h prior to experiments. WM35 cells were cultured in RPMI 1640 supplemented with 10% fetal bovine serum, 2 mM L-glutamine, 5 μg/ml insulin, and 50 U/ml penicillin and 50 μg/ml streptomycin at 37°C in a water-saturated atmosphere of 5% CO_2 _and 95% air.

### PCR

RNA was isolated using High Pure RNA Isolation Kit from Roche (Mannheim, Germany). For synthesis of cDNA SuperScript III from Invitrogen (Paisley, Scotland) was used and DynaZyme EXT from FinnZymes (Espoo, Finland) was used for the PCR-reactions. All steps were done according to the instructions given by the manufacturers. PCR reactions were performed by first heating the reaction mix to 94°C for 5 minutes. This was followed by 29 cycles of 30 seconds at 94°C, 60 seconds at the annealing temperature and elongation 60 seconds at 72°C. The primers, annealing temperatures, and product lengths were: S1P_1_, sense GGCTGGAACTGCATCAGTGCG, antisense GAGCAGCGCCACATTCTCAGAGC, 60°C, 223 bp; S1P_2_, sense CCGAAACAGCAAGTTCCACT, antisense CCAGGAGGCTGAAGACAGAG, 61°C, 197 bp; S1P_3_, sense AAGGCTCAGTGGTTCATCGT, antisense GCTATTGTTGCTGCTGCTTG, 61°C, 201 bp; S1P_4_, sense CCTTCAGCCTGCTCTTCACT, antisense AAGAGGATGTAGCGCTTGGA, 64°C, 223 bp; S1P_5_, sense AGGACTTCGCTTTTGCTCTG, antisense TCTAGAATCCACGGGGTCTG, 59°C, 201 bp.

### S1P-production assay

Cells (roughly 270 000 cells per 35-mm cell culture plate) were incubated over night in medium with serum replaced by 0.2% fatty acid-free BSA. Cells were then stimulated with agonist or vehicle together with [^3^H] sphingosine (~ 200,000 cpm) with fatty acid free BSA as carrier. Lipids were extracted by aspirating the culture medium and adding 500 μl of ice-cold methanol to the cells. Cells were scraped from the petri dishes and transferred to eppendorf tubes. The tubes were sonicated for 5 minutes and then centrifuged at 6,000 g for 10 minutes to remove cell debris. The supernatant was then transferred to glass vials. S1P was added to each sample for identification and the supernatant was evaporated. For measurements of secreted S1P, [^3^H]S1P was extracted from the medium as previously described previously [29]. Briefly, 2.2 ml of chloroform: methanol: HCl (50:50:1) was used to extract S1P from 900 μl medium. The organic phase was collected and evaporated. After re-dissolving in methanol the samples were spotted onto HPTLC plates and separated with butan-1-ol: acetic acid: water (3:1:1, v/v). S1P was stained with ninhydrin and spots were scraped and the formed [^3^H]S1P was counted using liquid scintillation. From a typical experiment the recovered counts of intracellular and secreted [^3^H]S1P at basal conditions were 449 ± 69 cpm and 175 ± 20 cpm, respectively in HeLa cells. Under similar conditions 225 ± 34 cpm was extracted from MEL-7 cells and 557 ± 61 cpm from the medium. WM35 cells had a basal cellular [^3^H]S1P of 213 ± 18 cpm and secreted 288 ± 55 cpm.

### Construction of a viral vector containing human SphK1 and transduction of HeLa cells

Human SphK cDNA was cloned and FLAG -tagged at the 3' end according to Pitson et al [30]. The SphK-FLAG fragment was PCR amplified by using primers with 5' *Mlu*I and 3' *Sal*I sites and cloned into the WPT-GFP lentiviral vector which had been digested with *Mlu*I and *Sal*I to remove the GFP gene. Lentiviral vectors expressing the SphK-FLAG construct were produced by transient three plasmid cotransfection into HEK 293T cells by using calcium phosphate precipitation. The three plasmid mixture consisted of 14.5 μg WPT-SphKFLAG, 8.3 μg pCMVΔR8.91 and 2.1 μg MD.G (all plasmids were a kind gift from Dr. D. Trono, Lausanne, Switzerland). The virus-containing media were harvested 48 hours later by filtering the media through 0.45 μm pore size filter and centrifuging at 16 000 g for 2.5 h at +4°C. The resulting pellets were resuspended in 200 μl serum free DMEM. For transduction HeLa cells were plated on 6-well plates (1 × 10^5 ^per well) and 24 hours later virus together with 8 μg/ml Polybrene was added at multiplicity of infection 10 and incubated for 6 hours after which time the medium was replaced with fresh medium.

### siRNA mediated knock down

The cells were grown to 90% confluency, and transfection was done with N-TER transfection reagent according to the manufacturer's protocol for serum-free transfection with slight modifications. The siRNA was added to the cells at a final concentration of 100 nM. Following a 24 hour incubation with the siRNA reagent, the medium was changed to fresh medium containing 0.2% Fatty acid free BSA. Following another 24 h incubation the cells were used for experiments.

### NF-κB activation assay

Cells grown on 60 mm petri dishes were harvested and pelleted in ice cold PBS. The cell pellet was quick-freezed and resuspended in 150 μl buffer containing 25% glycerol, 0.42 M NaCl, 1.5 mM MgCl_2_, 0.2 mM EDTA, 20 mM Hepes (pH 7.9), 0.5 mM DTT, and 0.5 mM PMSF. The extract was then centrifuged at 15,000 × g for 20 minutes at +4°C. The supernatant was collected and protein concentrations were determined. Two methods for assaying NF-κB (p65) DNA-binding activity were used. NFκB transactivation capacity was measured from the extracts either by using a Trans-AM NF-κB (p65) transcription factor assay kit (Active Motif, Carlsbad, CA) according to the manufacturers' instructions, or by electrophoretic mobility shift assay (EMSA). The results from the Trans-AM NF-κB (p65) transcription factor assay kit are presented as percent activation with the stimulated control set to 100% and the unstimulated control as 0%. This was made to compensate for between-experiment differences in NF-κB activation. For the EMSA experiments, the consensus NF-κB binding site (5'-AGCTTCAGAGGGGACTTTCCGAGAGG-3') was ^32^P-labeled. Protein-DNA complexes were separated on a native 4% polyacrylamide gel. The gel was dried and exposed to autoradiography film over night. Both methods measure NF-κB activity by detecting it binding to its consensus binding sequence.

### Apoptosis assay

Cells were assayed using an active caspase-3 detection kit from BD Pharmingen according to the manufacturers' instructions. In brief, cells were collected in eppendorf tubes, spun down and washed twice in 1 ml PBS. The cells were resuspended in 150 μl Cytofix/Cytoperm™ Solution. Following a 20 minute incubation on ice the cells were washed twice with 0.5 ml Perm/Wash™ Buffer. The cells were then incubated with the phycoerythrin-labelled antibody against active caspase-3 (1:20 in 100 μl Perm/Wash™ Buffer). The cells were washed once in 1 ml Perm/Wash™ Buffer. The cells were resuspended in 0.5 ml Perm/Wash™ Buffer and were then analyzed by flowcytometry using a BD FACScan and Cell Quest software.

### Western blotting

35 mm-petri dishes were washed once with cold HBSS, and scraped in 70 μl lysis buffer (10 mM Tris/HCl (pH 7.7), 150 mM NaCl, 7 mM EDTA, 0.5% NP-40, 0.2 mM PMSF, and 0.5 μg/ml leupeptin). Lysates were kept on ice for 15 minutes and were then centrifuged at 10,000 g for 15 minutes. 3 × Laemmli's buffer was mixed with the supernatant and the solution was heated to 95°C for 3 minutes. Proteins were separated by 10% SDS-PAGE and transferred onto a nitrocellulose membrane. The primary antibodies used were anti-Bcl-x_L _from Santa Cruz (CA, U.S.A.) and anti-Hsc70 from Stressgen (Victoria, Canada). HRP conjugated secondary antibodies were used, and bands were exposed on film by chemiluminescence.

### Statistics

Results are expressed as means ± SEM from a minimum of three independent experiments. Statistical analysis was made using Student's *t *test for paired observations. When three or more means were tested, one way ANOVA was performed followed by Dunnett's test for multiple comparisons against a single control. Statistical significance (p < 0.05) is denoted with *.

## Results

### S1P stimulates NF-κB dependent cell survival

**Figure 1:**
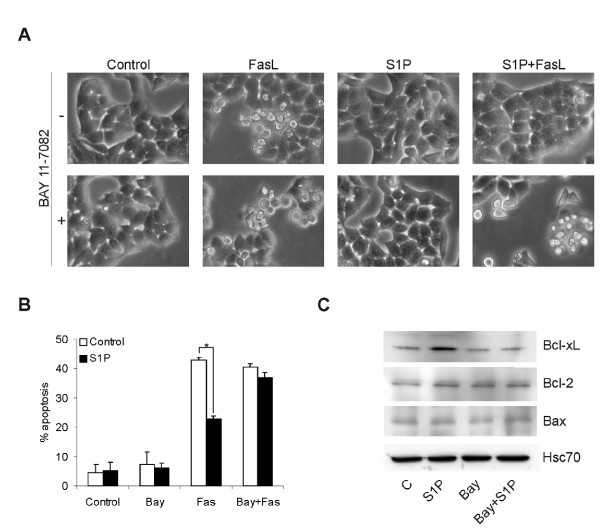
**S1P confers NF-κB dependent protection from death receptor induced apoptosis**. Figure **A **shows representative phase contrast pictures of HeLa cells treated ±5 μM Bay 11-7082 (NF-κB inhibitor) for 6 h, followed by addition of vehicle or 3 μM S1P for 8 hours. Apoptosis was then induced by addition of 50 ng/ml superFas ligand (Fas) for 16 hours. **B**. HeLa cells were treated as in A, and were fixed, stained for active caspase-3, and were analysed by FACS. The bars denote the mean ± SEM of at least three independent experiments (*, p < 0.05). **C**. Western blot showing Bcl-x_L_, Bcl-2 and Bax expression in cells pre-treated with 5 μM Bay 11-7082 or vehicle for 6 h, followed by stimulation with vehicle or 3 μM S1P for 12 hours. Hsc70 was used as a loading control. The results are representative of three independent experiments.

It has been firmly established that S1P is a cytoprotective agent. However, the downstream mechanisms are pleiotropic and have only been partially identified. To study the S1P-induced survival signaling pathways we first needed to identify a reproducible and quantifiable endpoint. NF-κB is a well-defined survival factor, and there are reports showing that S1P activates NF-κB by signaling through G-protein coupled S1P receptors [31-33]. NF-κB activation was therefore ideal to use as an endpoint for addressing S1P signaling pathways leading to survival. In our initial experiments, we could confirm the importance of NF-κB in S1P mediated survival signaling. S1P protected HeLa cells from Fas ligand-induced apoptosis in an NF-κB dependent manner (Figure 1A, B). S1P also induced an upregulation of the anti-apoptotic protein Bcl-xL which was blocked by the NF-κB inhibitor Bay 11-7082 (Figure 1C).

### S1P activates NF-κB through G protein coupled receptors

**Figure 2:**
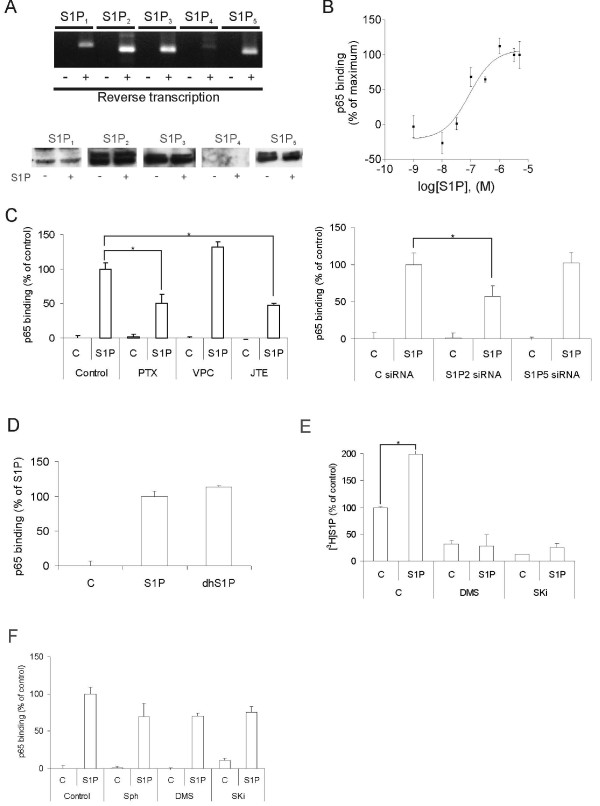
**S1P activates NF-κB through G protein coupled receptors**. **A**. The upper panel shows the presence of mRNAs encoding G protein coupled S1P receptors in HeLa cells. + and - denote whether the extracted RNA was reverse transcribed or not. The lower panel shows Western blots of the S1P receptors in HeLa cells stimulated ± 3 μM S1P for 30 minutes. **B**. Concentration-response curve for S1P-induced activation of NF-κB (p65). HeLa cells were stimulated with varying concentrations of S1P for 30 minutes. Protein extracts were analyzed by NF-κB (p65) ELISA. 5 μg of protein was used from each extract. **C**. HeLa cells were preincubated with 50 ng/ml PTX for 16 h, 10 μM VPC23019 for 30 minutes or 10 μM JTE013 for 30 minutes prior to stimulation with 3 μM S1P for 30 minutes (left panel), or treated with S1P_2 _or S1P_5 _siRNA for 48 h prior to S1P stimulation. Proteins were then extracted and NF-κB (p65)-activation was assayed by ELISA. **D**. HeLa cells were stimulated with either vehicle, 3 μM S1P or 3 μM dihydro-S1P for 30 minutes. 5 μg of protein from each extract was used for the NF-κB (p65) ELISA. **E**. HeLa cells were pre-incubated with either vehicle, 10 μM DMS or 10 μM SKi for 5 minutes. The cells were then stimulated with 3 μM S1P for 10 minutes in the presence of [^3^H]sphingosine. The bars show synthesized cellular [^3^H]S1P as percent of the unstimulated control. **F**. The cells were pre-incubated either with vehicle, 10 μM sphingosine, 10 μM DMS or 10 μM SKi for 5 minutes. Following a 30-minute stimulation with 3 μM S1P, the cells were harvested, lyzed and the DNA-binding activity of p65 was measured from 5 μg of protein extract. The data points and bars in panels B-F denote the mean ± SEM of at least three independent experiments (*, p < 0.05).

S1P induces most of its functions by activating G protein coupled S1P receptors, but an intracellular mode of action has been proposed to take place in some cases. We therefore addressed the site of action for S1P-induced NF-κB activation in HeLa cells. The receptors S1P_1-3 _and S1P_5 _were detectable by PCR and Western blot in HeLa cells (Figure 2A). S1P induced NF-κB activation with an EC_50 _of 88 nM, which is consistent with a G protein coupled receptor -mediated effect (Figure 2B). Furthermore, the S1P-induced NF-κB activation was attenuated by the G_i _inhibitor pertussis toxin, by the S1P_2 _antagonist JTE013, and in S1P_2 _siRNA treated cells (Figure 2C). The S1P_1/3 _antagonist VPC 23019 did not inhibit the S1P induced NFκB activation indicating that the effect is not dependent on S1P_1 _or S1P_3 _(Figure 2C). In addition, dihydro-S1P potently activated NF-κB (Figure 2D). Dihydro-S1P binds and activates G protein coupled S1P receptors and has often been used as a negative control for intracellular effects of S1P [34,35]. Taken together, these results show that the S1P induced activation of NF-κB is mediated through an extracellular mode of action. It is known that extracellular S1P can cause cells to increase their intracellular production of S1P [17,18]. To exclude the possibility that intracellularly produced S1P was responsible for the NF-κB response, we measured the activation of NF-κB in cells treated with sphingosine kinase inhibitors. The cells were stimulated with 3 μM S1P to saturate G-protein coupled S1P receptors and to achieve maximal NF-κB activation (see Figure 2B). Under these conditions, the sphingosine kinase inhibitors DMS and SKi effectively blocked both the basal and agonist-induced S1P-production (Figure 2E). However, the NF-κB activation induced by extracellular S1P was not significantly affected (Figure 2F). These observations are in line with earlier studies [31-33] showing that S1P-induced NF-κB -activation is mediated through GPCR's.

### NF-κB activation induced by exogenously added S1P is enhanced by S1P produced and secreted by the cells

**Figure 3:**
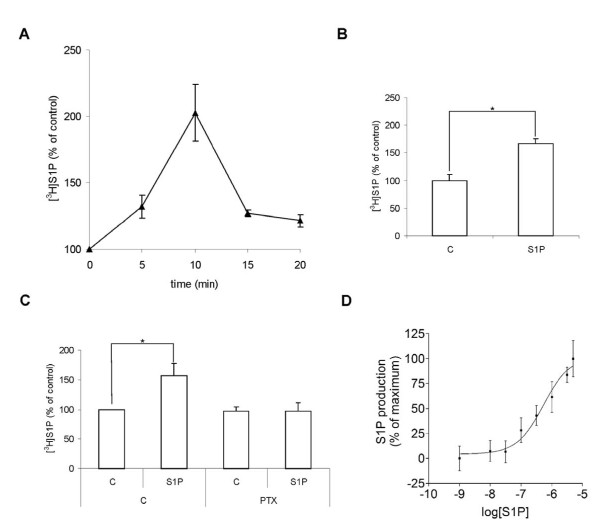
**S1P induces cellular S1P-production and -secretion through G_i _protein coupled receptors**. **A**. HeLa cells were exposed to either vehicle or 3 μM S1P for the indicated period of times in the presence of [^3^H]sphingosine. The amount of produced [^3^H]S1P is expressed as percent of that in vehicle-treated cells. **B**. Secreted [^3^H]S1P was extracted from the cell culture medium following a 10 minute stimulation with vehicle or 3 μM S1P, and was analyzed using thin layer chromatography and scintillation counting. **C**. The cells were treated with 50 ng/ml pertussis toxin or vehicle for 16 h. Cellular lipids were extracted following a 10 minute stimulation with 3 μM S1P or vehicle in the presence of [^3^H]sphingosine. The formed [^3^H]S1P was assayed using thin layer chromatography and scintillation counting. **D**. Concentration-response curve for S1P-induced S1P production. HeLa cells were stimulated with varying concentrations of S1P or vehicle together with [^3^H]sphingosine for 10 minutes. Cellular lipids were extracted and separated by HPTLC. The results are expressed as percent increased [^3^H]S1P production in S1P stimulated cells compared with control. The data points and bars in panels A-D denote the mean ± SEM of at least three independent experiments (*, p < 0.05).

**Figure 4:**
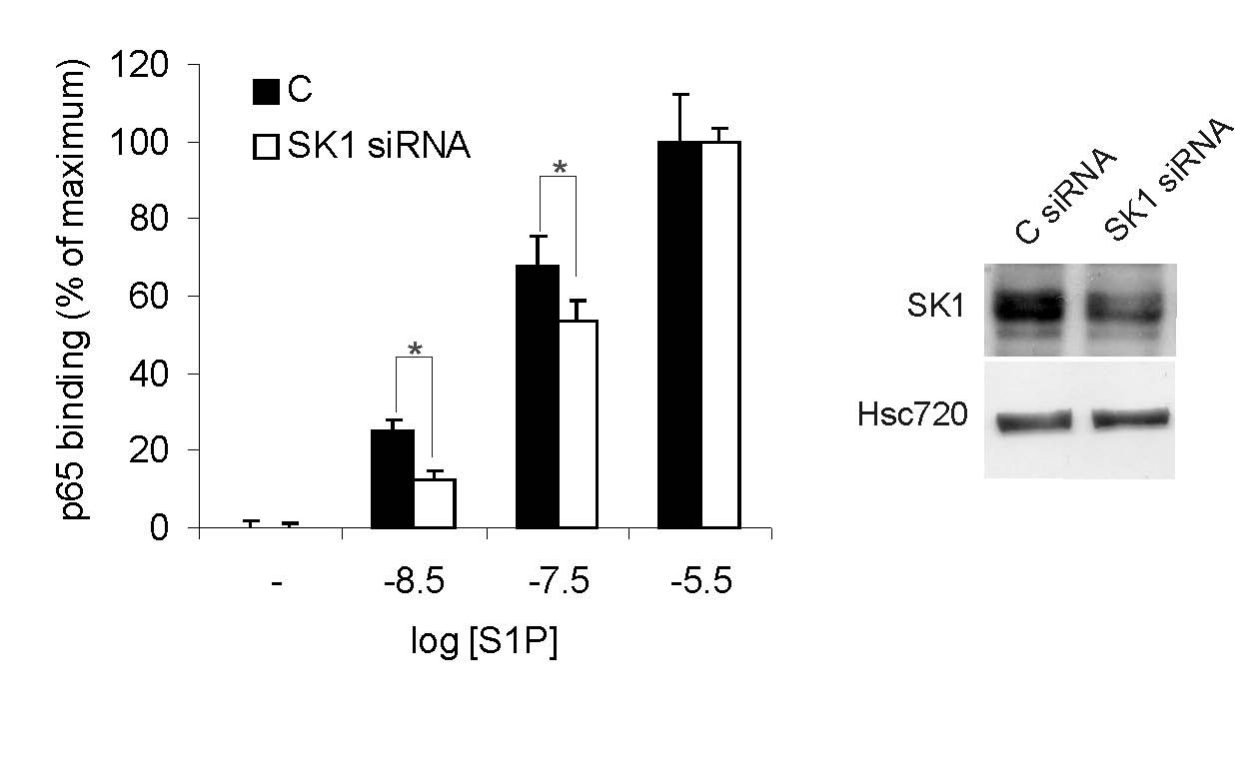
**NF-κB activation induced by exogenously added S1P is enhanced by S1P produced by the cells**. Cells transfected with control siRNA or SK1 siRNA were treated with the indicated concentrations of S1P for 30 minutes and were analyzed for active NF-κB (p65) using ELISA. The panel to the right shows representative western blots of control and SphK1 siRNA treated cells. The bars denote the mean ± SEM of at least three independent experiments (*, p < 0.05).

Extracellularly added S1P induced a transient increase in cellular S1P-production (Figure 3A), accompanied by an increased secretion of S1P (Figure 3B). The S1P-induced S1P-production was inhibited by pertussis toxin, suggesting dependence on GPCR-signaling (Figure 3C). The S1P induced S1P-production displayed a saturable concentration response curve, with a noticeable response following stimulation with 100 nM extracellular S1P (Figure 3D). There are now several reports showing that intracellularly produced S1P can be exported and act on cells in an autocrine or paracrine fashion [24-26]. Since extracellular S1P stimulated the cells' own production and secretion of S1P, we hypothesized that this could constitute a self amplifying signaling mechanism. When cells are stimulated with high concentrations of extracellular S1P, such a mechanism will not be noticeable, since all S1P-receptors will be saturated and not affected by any additional S1P that is secreted from the cells. However, if non-saturating concentrations of S1P are added to the cells, endogenously produced and secreted S1P might have an enhancing effect. To test this hypothesis we did concentration-response measurements for S1P-induced NF-κB activation with or without sphingosine kinase inhibition. A higher concentration of extracellular S1P was needed to achieve half-maximal NF-κB-activation in SphK1 siRNA treated cells compared to control cells (Figure 4). In additional experiments we found that there was a shift in the EC_50 _value from 74 nM in control cells (comparable to the 88 nM in figure 2B) to 158 nM in cells treated with 10 μM DMS. This shift is large enough to be significant for G-protein signaling, i.e. at the EC_50 _concentration, approximately half of the activating S1P is of cellular origin. Taken together, these results are in support of a model where S1P signaling is amplified through an autocrine feed-forward loop.

### Effect of prolonged overexpression of SphK on NF-κB activation and S1P synthesis

**Figure 5:**
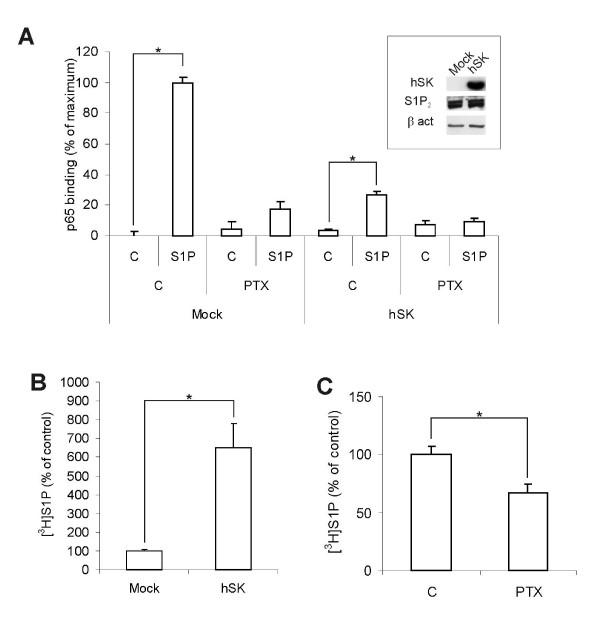
**Effect of prolonged overexpression of SphK on NF-κB activation and S1P synthesis**. **A**. Control cells and cells overexpressing SphK (hSK) were pretreated ± 50 ng/ml pertussis toxin for 16 h before stimulation with vehicle or 3 μM S1P for 30 minutes. Proteins were extracted and NF-κB (p65)-activation was assayed by ELISA. The inset shows Western blots of 10 μg of protein from mock transduced or SphK1 overexpressing HeLa cells. The cell extracts were probed for sphingosine kinase and S1P_2_. **B**. [^3^H]S1P was extracted from the mock- or SphK transduced cells following a 10-minute incubation with [^3^H]sphingosine, and was analyzed using thin layer chromatography and scintillation counting. **C**. The SphK overexpressing cells were treated with 50 ng/ml pertussis toxin or vehicle for 16 h. Secreted [^3^H]S1P was extracted from the medium following a 10-minute incubation with [^3^H]sphingosine. The formed [^3^H]S1P was assayed using thin layer chromatography and scintillation counting. The bars in panels A-C denote the mean ± SEM of at least three independent experiments (*, p < 0.05).

Next, we performed studies on cells overexpressing SphK1. A lentivirus based system was utilized for overexpressing human SphK1 in HeLa cells. Extracellular S1P efficiently activated NF-κB in mock-transduced cells. This activation was blocked by G_i _protein inhibition, confirming the dependence on GPCR-signaling. Interestingly, in cells overexpressing SphK1, exogenous S1P only weakly activated NF-κB (Figure 5A). The cellular level of the receptor S1P_2 _was not affected by SphK1 overexpression (Figure 5A inset), suggesting that this observation is likely explained by homologous desensitization of S1P_2 _receptors without subsequent receptor degradation as shown by Jolly et al [36]. Since the NF-κB activation was mediated through G protein coupled S1P-receptors and independent of an intracellular action of S1P, these results seem to confirm that autocrine S1P modulates NF-κB signaling. Cells overexpressing SphK had an elevated basal S1P production of 650 ± 130% compared to control cells (Figure 5B). Treating the SphK overexpressing cells with pertussis toxin for 16 h lead to a significant decrease in the amount of secreted S1P (Figure 5C), which is in line with an autocrine feed-forward loop upholding the production and secretion of S1P.

### Signaling cascades regulating S1P-induced S1P-production and S1P-induced activation of NF-κB

**Figure 6:**
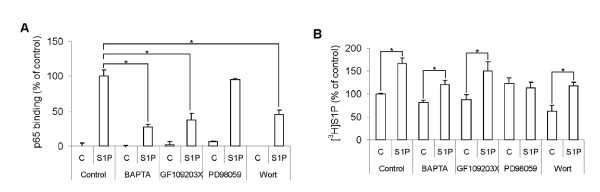
**Signaling cascades regulating S1P-induced S1P-production and S1P-induced activation of NF-κB**. **A**. NF-κB-activation induced after stimulating HeLa cells for 30 minutes with 3 μM S1P. The cells were pre-treated with 10 μM BAPTA-AM for 30 minutes, 50 μM PD98059 for 30 min, 1 μM GF109203× for 5 min or 30 nM Wortmannin for 5 minutes. **B**. The cells were treated with inhibitors the same way as in A. Following a 10-minute stimulation with 3 μM S1P or vehicle together with [^3^H]sphingosine, lipids were extracted and the produced [^3^H]S1P was measured by thin layer chromatography and scintillation counting. The bars in A and B denote the mean ± SEM of at least three independent experiments (*, p < 0.05).

PKC, phosphatidyl-inositol 3-kinase (PI3K), mitogen activated protein kinase (MAPK) and Ca^2+ ^are effectors known to act downstream of S1P-receptors. We therefore compared the roles of these signaling components in S1P-induced activation of NF-κB and in S1P-induced S1P-production. The intracellular Ca^2+ ^chelator BAPTA-AM, the PKC inhibitor GF109203× and the PI3K inhibitor wortmannin attenuated the S1P-induced NF-κB-activation, whereas the MAPK-kinase (MEK) inhibitor PD98059 was without an effect (Figure 6A). The S1P-induced increase in cellular S1P-production was completely blocked by PD98059, slightly reduced by Ca^2+ ^chelation, and not significantly affected by neither GF109203× nor wortmannin, (Figure 6B). These results are in accordance with previous findings that ERK1/2 and Ca^2+ ^are important factors for activating and translocating SphK to the plasma membrane respectively [19,20]. These results illustrate that S1P utilizes different signaling pathways to induce activation of NF-κB and to stimulate S1P-production.

### S1P-induced secretion of S1P in the malignant tumor cell lines MEL-7 and WM35

**Figure 7:**
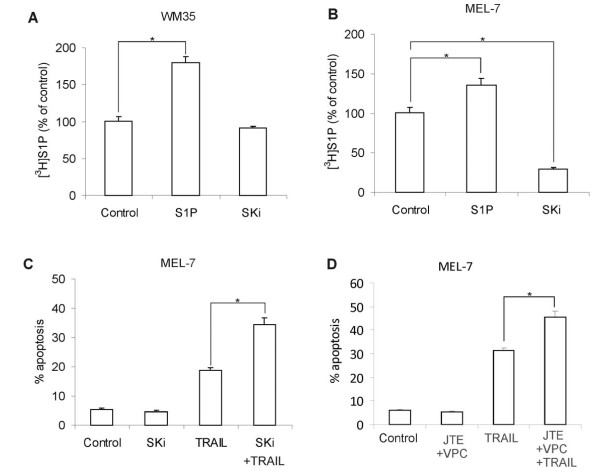
**S1P-induced secretion of S1P in the malignant tumor cell lines MEL-7 and WM35**. WM35 (**A**.) or MEL-7 (**B**.) cells were exposed to either vehicle, 3 μM S1P or 10 μM SKi for 10 minutes in the presence of [^3^H]sphingosine. Secreted [^3^H]S1P was then extracted from the cell culture medium and analysed by HPTLC. **C**. MEL-7 cells were treated with 10 μM SKi or vehicle for 4 h. Apoptosis was then induced by addition of 100 ng/ml Tumor necrosis factor-related apoptosis-inducing ligand (TRAIL) for 4 h. **D**. MEL-7 cells were treated with vehicle or 10 μM JTE013 and VPC23019 for 4 h. Apoptosis was induced by addition of TRAIL for 4 h, and cells positive for cleaved caspase-3 was measured using FACS. The bars in panels A-D denote the mean ± SEM of three independent experiments (*, p < 0.05).

We used the two malignant melanoma cell lines MEL-7 and WM35 to test whether our proposed self-amplifying autocrine loop is also present in other than HeLa cells. In both cell lines, the secretion of S1P was enhanced by the addition of exogenous S1P (Figure 7A, B). This suggests that the described self-amplifying autocrine signaling is common also in other types of tumor cells. Since the MEL-7 cells displayed a higher basal secretion of S1P (results not shown), we tested whether sphingosine kinase was involved in survival signaling in this cell line. We found that inhibition of sphingosine kinase *per se *did not render the tumor cells apoptotic, but sensitized them to apoptosis induced by TRAIL (Figure 7C), a selective inducer of death in many transformed cells but not in most normal cells. To address whether this effect was due to secreted S1P, we repeated the experiment in cells treated with JTE013 and VPC23019 to block signaling via S1P_1-3 _(Figure 7D). Similar to what was observed in cells treated with SKi, the receptor antagonists alone did not affect apoptosis, but increased the potency of TRAIL to induce cell death. This indicates that the observed effect is due to S1P secretion rather than an intracellular mode of action. Down-regulation of the self-amplifying S1P signaling in combination with TRAIL treatment is therefore potentially useful in cancer therapy.

## Discussion

Our knowledge regarding how sphingolipids regulate cell survival or death has been rapidly increasing since the concept of a sphingolipid "rheostat" was first introduced. S1P has emerged as a central bio-active lipid with both intracellular and extracellular actions. There is some controversy surrounding sphingolipids and their sites of action in survival signaling. Since one type of sphingolipid may be rapidly converted into another sphingolipid with opposite effects on survival, it has proven difficult to determine to which extent each lipid-type is responsible for the outcome of the signaling. In the present report we show that S1P antagonizes death receptor-induced apoptosis in HeLa cells by acting through G protein-coupled S1P receptors and activating the transcription factor NF-κB. S1P_2 _was at least in part responsible for activating NF-κB, but it seems likely that S1P promotes survival through simultaneous activation of several signaling cascades. It has previously been shown that S1P may activate NF-κB through S1P_2 _and S1P_3 _in a PLC/PKC-dependent manner [32]. We could confirm that the activation was dependent on PKC and on S1P_2 _in HeLa cells. The PI3K inhibitor wortmannin also attenuated the S1P-induced activation of NF-κB, in accordance with what has been shown in vascular smooth muscle cells [37].

It was first shown by Meyer zu Heringdorf et al [17], and later by us [18] that S1P may stimulate its own intracellular synthesis, but whether this also leads to an increase in S1P-secretion has not been previously investigated. We show here that S1P not only stimulates the production of intracellular S1P, but also its secretion. Based on concentration response curves for the S1P-induced NF-κB activation in figure 4, we conclude that the secreted S1P is an important contributing factor in S1P signaling. The S1P-induced S1P-synthesis was sensitive to MEK-inhibition, but not to inhibition of PKC or PI3K. The opposite was true for S1P-induced NF-κB-activation, which suggests that these two mechanisms can be regulated independently of each other. This was further illustrated by the fact that the S1P_2 _antagonist JTE013, and S1P_2 _knockdown attenuated the S1P-induced activation of NF-κB, while the S1P-induced S1P-production was unaffected. In conclusion, the results we present here lend support to a novel feed-forward mechanism, with S1P stimulating its own synthesis and secretion. The secreted S1P may then protect the tumor cells from death receptor-induced apoptosis by contributing to NF-κB activation. An important consequence of the autocrine feed-forward signaling is that an initially small increase in the cells' S1P-production can be considerably amplified. The secreted S1P provides protection for the tumor cell itself, and may also activate a similar feed-forward mechanism in surrounding cells. In addition to stimulating cell survival, S1P also induces cell proliferation [38] and angiogenesis [39]. These factors are crucial for tumor development and metastasis.

## Conclusions

S1P protects HeLa cells from FasL-evoked apoptosis in an NF-kB-dependent manner. We propose that this effect is mediated by a cytoprotective mechanism involving an amplification loop where S1P stimulates its own production and secretion by activating G protein coupled S1P-receptors. It is likely that the mechanism presented here is important for tumor progression.

## Abbreviations

S1P: sphingosine-1-phosphate; NFκB: nuclear factor κB; SphK: Sphingosine kinase; DMS: N,N-dimethylsphingosine; SKi: 2-(*p*-Hydroxyanilino)-4-(*p*-chlorophenyl)thiazole.

## Authors' contributions

TB, JEE and KT planned the experiments; TB, NB, AM and CL performed experiments, JPS contributed with analytical methods and reagents; TB, JEE and KT wrote the paper. All Authors read and approved the final manuscript.

## Acknowledgements

This work was supported by grants from the Oskar Öflund Foundation (TB), the Sigrid Juselius Foundation (KT, JEE), The Liv och Hälsa Foundation (KT), Svenska Kulturfonden (KT) and the Academy of Finland (TB, JEE, KT). The study was done as a part of the Receptor Structure and Function program at the University of Turku, Åbo Akademi University and the National Public Health Institute, and as a part of the Center of Excellence in Cell stress consortium at Åbo Akademi University. Tomas Blom, Annika Meinander and Nina Bergelin were supported by the Turku Graduate School of Biomedical Sciences during this work. The funding bodies had no role in in study design, data collection, analysis, interpretation, in writing the manuscript, or in the decision to submit the paper for publication.
